# Functional and Structural Analysis of the Internal Ribosome Entry Site Present in the mRNA of Natural Variants of the HIV-1

**DOI:** 10.1371/journal.pone.0035031

**Published:** 2012-04-04

**Authors:** Maricarmen Vallejos, Felipe Carvajal, Karla Pino, Camilo Navarrete, Marcela Ferres, Juan Pablo Huidobro-Toro, Bruno Sargueil, Marcelo López-Lastra

**Affiliations:** 1 Laboratorio de Virología Molecular, Instituto Milenio de Inmunología e Inmunoterapia, Centro de Investigaciones Médicas, Escuela de Medicina, Pontificia Universidad Católica de Chile, Santiago, Chile; 2 Centro de Envejecimiento y Regeneración, CARE, Departamento de Fisiología, Facultad de Ciencias Biológicas, Pontificia Universidad Católica de Chile, Santiago, Chile; 3 Laboratorio de Infectología, Facultad de Medicina, Pontificia Universidad de Chile, Santiago, Chile; 4 Centre national de la recherche scientifique, Unité Mixte de Recherche 8015, Laboratoire de cristallographie et RMN Biologique, Université Paris Descartes, Paris, France; Pohang University of Science and Technology, Republic of Korea

## Abstract

The 5′untranslated regions (UTR) of the full length mRNA of the HIV-1 proviral clones pNL4.3 and pLAI, harbor an internal ribosomal entry site (IRES). In this study we extend this finding by demonstrating that the mRNA 5′UTRs of natural variants of HIV-1 also exhibit IRES-activity. Cap-independent translational activity was demonstrated using bicistronic mRNAs in HeLa cells and in *Xenopus laevis* oocytes. The possibility that expression of the downstream cistron in these constructs was due to alternative splicing or to cryptic promoter activity was ruled out. The HIV-1 variants exhibited significant 5′UTR nucleotide diversity with respect to the control sequence recovered from pNL4.3. Interestingly, translational activity from the 5′UTR of some of the HIV-1 variants was enhanced relative to that observed for the 5′UTR of pNL4.3. In an attempt to explain these findings we probed the secondary structure of the variant HIV-1 5′UTRs using enzymatic and chemical approaches. Yet subsequent structural analyses did not reveal significant variations when compared to the pNL4.3-5′UTR. Thus, the increased IRES-activity observed for some of the HIV-1 variants cannot be ascribed to a specific structural modification. A model to explain these findings is proposed.

## Introduction

Initiation of protein synthesis in the eukaryotic cell is a complex process that leads to the assembly of the 80S ribosome at the start codon of the mRNA [1], [2], [3]. In eukaryotic cells two mechanisms for recruiting and positioning ribosomes on the mRNA have evolved [1], [2], [3]. The primary mechanism involves the recognition of the 5′ cap structure (m^7^GpppN, where N is any nucleotide) on the mRNAs by eukaryotic translation initiation factors (eIFs) that deliver the 40S ribosomal subunit. Upon recruitment to the mRNA, the 40S ribosomal subunit scans downstream along the 5′ untranslated region (UTR) until the initiation codon is encountered. Alternatively, an RNA structure termed the internal ribosome entry site (IRES) can drive 40S ribosomal subunit recruitment and positioning on the mRNA either at or upstream of the start codon [2], [4], [5].

The 5′ UTR of the full length or genomic RNA of the human immunodeficiency virus type-1 (HIV-1), prototype member of the lentivirus genus of the *Retroviridae* and the etiologic agent of AIDS, plays key roles during the viral life cycle [6]. The study of the mechanism of translation exhibited by HIV-1 genomic RNA revealed that the synthesis of the viral structural protein Gag can be initiated both through the canonical cap-dependent mechanism [7], [8], [9], or by the alternative IRES-dependent mechanism [9], [10], [11], [12], [13], [14]. The capped and polyadenylated HIV-1 genomic RNA has been described to harbor two IRESes, the first within the mRNA’s 5′UTR (here referred to as the HIV-1 IRES) [10], [14], and the second within the Gag open reading frame (referred to here as the HIV-1 *gag* IRES) [11], [12], [15]. The observed conservation of both cap- and IRES-dependent mechanisms of translation initiation among primate lentiviruses, coupled with the redundancy this provides, suggests that initiation of proteins synthesis is a key process during the viral life cycle [9], [12], [13], [16].

Since its first characterization [10] a number of additional reports have addressed various aspects concerning the HIV-1 IRES [9], [13], [14], [17], [18], [19], [20]. One recognized caveat, however, of these and other reports concerning the mechanism of HIV-1 translation initiation is that findings are based upon the study of the 5′UTR sequence recovered from only two infectious clones of HIV-1, namely pNL4.3 and pLAI [10], [14], [18], [19]. The existence and relevance of IRES activity in the context of natural HIV-1 variants remains a matter of controversy and debate [7], [16]. In this study we demonstrate that the 5′UTRs of viral RNAs isolated from HIV-1 infected individuals show a capability to drive cap-independent translation initiation similar to that observed for the 5′UTRs of clones pNL4.3 and pLAI [10], [14], [18], [19].

## Results

### The HIV-1 5′UTR Isolated from Natural Viral Variants Drives Translation when in the Context of a Bicistronic mRNA

**Figure 1:**
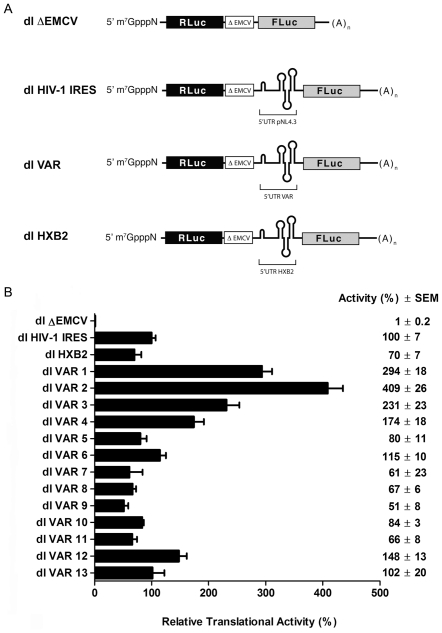
Translational activity of the natural HIV-1 5′UTR variants isolated from clinical samples. (A) Schematic representation of the bicistronic vectors (mRNAs) used in this study. Vectors dl ΔEMCV and dl HIV-1 IRES have been previously described [10], [25]. The HIV-1 5′UTRs recovered from the HXB2 infectious clone or from RNA isolated from clinical samples were cloned into dual luciferase bicistronic (dl) vectors harboring *Renilla* (RLuc) and Firefly (FLuc) luciferases as reporter genes to generate the dl VAR and dl HXB2 vectors. The dl VAR vectors were arbitrarily numbered 1-13. The 5′UTR recovered from the HIV-1 infectious clone pNL4.3, dl HIV-1 IRES, was used as the reference IRES [10], while the RLuc/FLuc bicistronic vector harboring a defective encephalomyocarditis virus (ΔEMCV) IRES, dl ΔEMCV, was used as a negative control [10], [25]. (B) Bicistronic vectors were transfected into HeLa cells, which were processed 24 h thereafter. RLuc and FLuc activities were measured and the [(FLuc/RLuc)] ratio was used as an index of IRES activity, with the [(FLuc/RLuc)] ratio of the dl HIV-1 IRES arbitrarily defined as 100%. Values are the means +/- SEM from three independent experiments.

At present, IRESes are defined solely by functional criteria [21,22]. The presence or absence of IRES activity within mRNAs of natural HIV-1 variants (VAR) was therefore evaluated using a bicistronic mRNA approach similar to that described by Brasey et al. (2003) and Gendron et al. (2011). To generate bicistronic VAR vectors (Fig. 1A), natural variant 5′UTR sequences (nucleotides 1-336 with respect to HIV-1 clone pNL4.3; AF 324493) were recovered from randomly pooled viral RNA extracts and cloned into a dual luciferase (dl) reporter construct containing an upstream *Renilla* luciferase gene (RLuc) and a downstream firefly luciferase gene (FLuc) [10]. All plasmids were sequenced (Macrogen Corp, Rockville, MD, USA). Variant HIV-1 5′UTR sequences were aligned against the 5′UTR of pNL4.3, used in this study as the reference IRES [10]. Thirteen dl VAR vectors arbitrarily numbered dl VAR 1 to dl VAR 13 were randomly selected to continue the study. The sequence differences between the VAR 5′ UTRs and pNL4.3 are highlighted in Table 1. As an additional control we also constructed the bicistronic vector dl HXB2 that features the 5′UTR of the HIV-1 clone HXB2 (recovered from bicistronic constructs described in [11]), the laboratory adapted version of HIV-LAI, in the intercistronic space (Fig. 1A). Plasmids (200 ng) were then transfected into HeLa cells using the JetPei system (Polyplus-transfection SA**,** Illkirch, France) following well established protocols [23]. After 24 h total proteins were recovered and the FLuc and RLuc activities were measured as described in material and methods. In common with previous studies [10,18,19,23,24], the FLuc/RLuc ratio was used as the readout of IRES activity expressed as relative translation activity (RTA), with the mean translation efficiency of the reference IRES (pNL4.3, dl HIV-1 IRES vector) arbitrarily set at 100% (+/- standard deviation). The dl ΔEMCV vector containing the ?EMCV sequence in the intercistronic space was used as a negative control (Fig. 1A) [10,25]. In agreement with the report of Gendron et al. (2011) the 5′UTR of the HXB2 infectious clone of HIV-1 exhibited IRES activity (Fig. 1B), confirming that cap-independent translation initiation is not restricted to the 5′UTR of the HIV-1 clone pNL4.3. Additionally, figure 1B shows that when in the context of a bicistronic mRNA all VAR 5′UTRs are capable of driving protein synthesis. However, translational activity of VAR-IRESes varied considerably, from approximately 50% to up to 400% RTA (Fig. 1B). The residual activity of the dl ΔEMCV RNA, lacking an IRES altogether, is 1% relative to the control dl HIV-1 IRES (not subtracted from the values presented in figure 1B). Collectively, these observations are in concordance with reported cap-independent translational activity by 5′UTR sequences of other members of the *Retroviridae* and some retroelements [10,14,23,26,27,28,29,30,31,32,33,34,35,36,37], and strongly support the notion that the 5′UTRs of all natural variants of HIV-1 recovered from clinical samples harbor active IRES elements. Additionally, the observed variability in RTA and, by inference IRES activities is difficult to ascribe to anything other than to 5′UTR sequence variability between clones (Table 1). Interestingly, yet in agreement with previous reports, careful comparative analysis of the variant sequences described herein reveals that translational activity from the HIV-1 IRES is remarkably forgiving of the introduction of mutations within its primary sequence [14,20].

**Table 1:** 

Variant (VAR)	Nucleotide changes with respect to pNL4.3 (AF 324493)
**1**	C23U, A46G, C95U, A133G, 149InsUAAUACU, C152G, 156DelInsAGAA, A161C, G166A, A168G, U200C, G202A, C207U, G217A, G224A, 256InsA, G257A, A263G, 301DelAA, A305U
**2**	C23U, A46G, C95U, A133G, 149InsUAAUACU, C152G, 156InsAGAA, A161C, G166A, C207U, G217A, G224A, 256InsA, A263G, G279A, A286G, 301DelAA, A305U
**3**	C23U, A46G, A47G, U86C, C95U, A133G, 149InsUAAUACU, C152G, 156DelInsAGAA, A161C, G166A, C207U, G217A, G224A, 256InsA, G257A, A263G, G279A, 301DelAA, A305U
**4**	A46G, C95U, A96C, A161C, A215G, G217A, G224A, A227C, C233U, G265A, C300U, A301G, A305U
**5**	A302U, A303C, A304C, A305U
**6**	U100C
**7**	C316U
**8**	A314G
**9**	U155C, A209G, G283A, 286DelA
**10**	A73G, U155C, A209G, G283A, 286DelA
**11**	A34G, G148C, A302U, A303C, A304C, A305U, G318A
**12**	G11A, C23U, U24A, A47G, C95U, A96C, C152A, U155G, 156InsU, A161C, 165DelU, G167A, U201C, A214U, 217DelG, A227C, C258U, 302DelAA, A304U
**13**	C23U, A47G, C95U, A96C, C152A, U153C, U155G, 156InsU, A161C, 165DelU, G167A, U201C, A214U, 217DelG, A227C, C258U, A301G, 303InsAAAAU, A304U
**HXB2**	C95U, A96C, U213G, A214G, G217A, A227C

Del: Deletion; Ins: Insertion.

### FLuc is Expressed from the Second Cistron in the Bicistronic mRNAs

**Figure 2:**
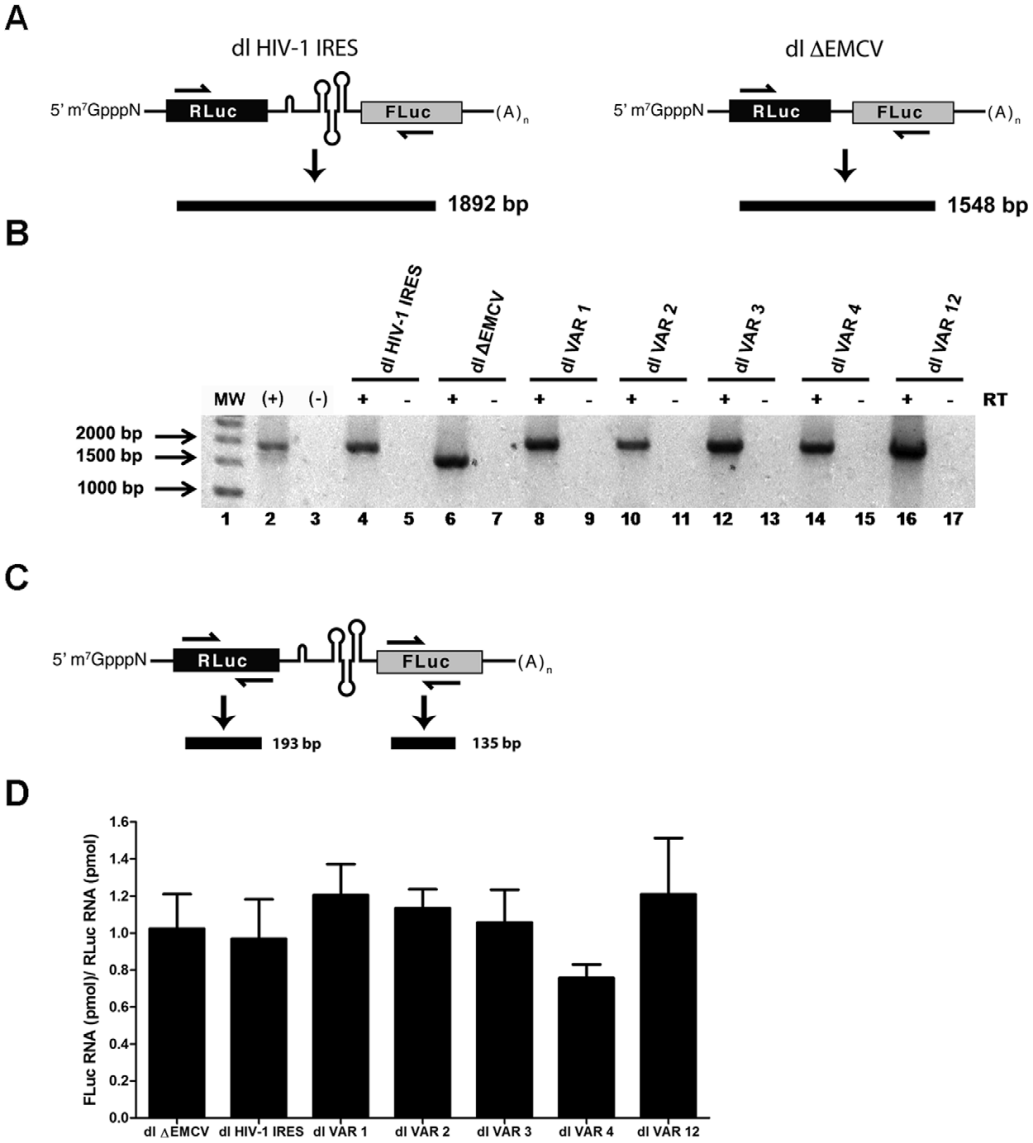
The full length bicistronic RNA is expressed from the dl VAR vectors. (A) Schematic representation of the experimental procedure to detect the full length bicistronic mRNA showing the primers and the size of the expected amplicons [23]. (B and D) HeLa cells were transfected with 200 ng of the dl HIV-1, dl ΔEMCV, or the different dl VAR plasmids. Total RNA was extracted from transfected cells and quantified. (B) Extracted RNA (3 µg) was used as template in a one-step RT-PCR designed to specifically detect the bicistronic RNAs (lanes 4, 6, 8, 10, 14, and 16). To assay for DNA contamination the same reaction was conducted in the absence of reverse transcriptase (lanes 5, 7, 9, 11, 13, 15, 17). *In vitro* transcribed dl HIV-1 IRES RNAs (lane 2) and water (lane 3) were included as RT-PCR controls. (C) Schematic representation of the experimental procedure to detect the RLuc-RNA and FLuc-RNA showing the primers and the size of the expected amplicons (D) Total RNA (200 ng) extracted from transfected HeLa cells was used as template in parallel RT-qPCR reactions designed to specifically detect the RLuc or FLuc containing RNAs. The RNA-FLuc concentration (pmol)/RNA-RLuc (pmol) ratio was calculated. Values are the means +/- SEM from three independent experiments (each RNA sample was amplified in three independent reactions).

Studies to identify internal initiation in isolated viral or cellular RNA regions utilizing the transfection of bicistronic reporter plasmids as described above have seen strong criticism [38], [39]; a commonly cited caveat to the experimental approach being false-positive IRES activity attributable to alternative splicing or cryptic promoter activity [38], [39]. In view of the sequence disparity between the natural variants and the reference pNL4.3-IRES [10], we undertook to verify the presence of the full length bicistronic RNA in cells transfected with the dl VAR plasmids (Table 1). VAR IRESes that exhibited activity similar to or below the reference pNL4.3-IRES, were not included in this analysis. Total RNA was extracted from cells previously transfected with dl VAR1, dl VAR2, dl VAR3, dl VAR4, or dl VAR12, and analyzed by RT-PCR as described by us and others (Fig. 2A) [23], [40], [41], [42]. Our results indeed confirmed the presence of full length bicistronic mRNA for the dl ΔEMCV, the dl HIV-1 IRES, and all tested dl VAR constructs (Fig. 2B). Even though results are not quantitative nor do they rule out the presence of other RNA species, they confirm, in all cases, the expression of the full-length bicistronic mRNA (Fig. 2B). No product was observed when the PCR reaction was conducted without a previous step of reverse transcription confirming the absence of DNA contamination in the RNA preparation (Fig. 2B). Next we quantified the amount of bicistronic mRNA by individually amplifying RLuc or FLuc using a quantitative RT-qPCR assay (Fig. 2C). For this total RNA extracted from cells transfected with dl ΔEMCV, dl HIV-1 IRES, dl VAR1, dl VAR2, dl VAR3, dl VAR4, or dl VAR12, was amplified in parallel reactions using primers specifically designed to recognize RLuc or FLuc (Fig. 2C). The RNA concentration (pmol) obtained by quantifying the FLuc cistron was divided by the RNA concentration (pmol) obtained by quantifying RLuc cistron (Fig. 2D). Results show that for the different RNAs the RNA-FLuc/RNA-RLuc ratio was close to one; dl ΔEMCV (1.0), dl HIV-1 IRES (0.97), dl VAR1 (1.2), dl VAR2 (1.1), dl VAR3 (1.0), dl VAR4 (0.75), or dl VAR12 (1.2) (Fig. 2D). One exception could however be VAR4 as the results revealed a slight, yet not significant, increase in bicistronic RNA when RLuc cistron was quantified (RNA-FLuc/RNA-RLuc ratio equal to 0.75). In despite of this observation, in general and when taking together, results presented in figures 2B and 2D suggest that only one RNA species expressing RLuc and FLuc is generated.

**Figure 3:**
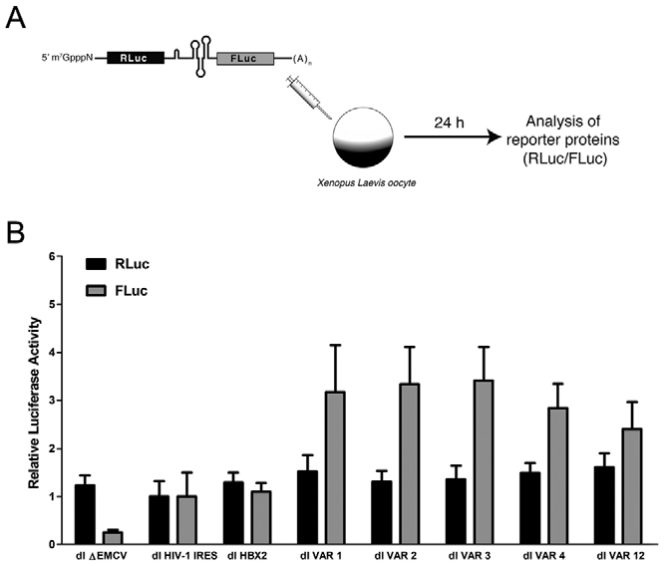
Assessment of IRES activity present within the HIV-1 VAR 5′UTR in *Xenopus laevis* oocytes. (A) Schematic representation of the experimental procedure. (B) Capped and polyadenylated RNA corresponding to the dl ?EMCV [10], [25], dl HIV-1 IRES [10], [19], [20], dl HBX2 (pLAI), or the different dl VAR 5′UTR (VAR 1, VAR 2, VAR 3, VAR 4, VAR 12) vectors (6.25 ng) were microinjected (50 nl final volume) into *Xenopus laevis* oocytes as previously described [19], [20], [23]. Oocytes were harvested 24 hr after microinjection, processed, and Renilla luciferase (RLuc) and Firefly luciferase (FLuc) activities were determined as described in Materials and Methods. Data are expressed as relative luciferase activities, the RLuc and FLuc activities of the dl HIV-1 IRES, the control bicistronic RNA harboring the 5′UTR of pNL4.3 in the intercistronic region between RLuc and FLuc [10], was arbitrarily set at 1. Each value is the mean +/- SEM from at least 3 oocytes.

Next we evaluated if this bicistronic mRNA could indeed drive cap- and IRES dependent translation initiation. After several unsuccessful results using RNA transfection assays in cells, we decided to address this question in *Xenopus laevis* oocytes, an experimental system that has proven useful for the study of viral IRESes [19], [20], [23], [43], [44]. I*n vitro* synthesized, capped and polyadenylated RNAs corresponding to the dl ΔEMCV, dl HIV-1 IRES, and the dl VAR vectors were microinjected into the cytoplasm of *Xenopus laevis* oocytes (Fig. 3A). RLuc and FLuc activities were measured 24 h post-injection (Fig.3A) as standardized in previous studies [19], [20], [23]. All values were expressed relative to the RLuc or FLuc activity obtained with the control dl HIV-1 IRES RNA [10], which was arbitrarily set to 1 (Fig. 3B). The RLuc levels from the dl HIV-1 IRES, and the dl VAR mRNAs were comparable, suggesting not only that cap-dependent translation initiation was functional in oocytes, but also confirming that similar amounts of RNA were microinjected in all cases (Fig. 3B). As described in previous studies [19], [20], [23], a low level of expression from the second cistron of the dl ΔEMCV reporter RNA, considered to be background, was also observed (Fig. 3B). FLuc from all the tested dl VAR RNAs was expressed well in excess of dl ΔEMCV background levels (Fig. 3.B), demonstrating that in the context of a bicistronic RNA the VAR 5′UTRs are certainly capable of driving cap-independent translation in *Xenopus laevis* oocytes. Again (see Fig.1) the translational activity of the VAR 5′UTRs was greater than that of the control pNL4.3 5′UTR, while the IRES activities of the HXB2 and pNL4.3 5′UTR were comparable (Fig. 3.B). Findings also suggest that RNA splicing is not a requirement for the expression of the FLuc reporter in *Xenopus laevis* oocytes. As an additional control dl ΔEMCV, dl HIV-1 IRES, and dl VAR DNA plasmids were microinjected into the cytoplasm of *Xenopus laevis* oocytes. In all cases neither RLuc nor FLuc activity could be detected, eliminating the possibility of *de novo* RNA synthesis from a DNA template (data not shown). Together, these data provide substantial evidence supporting the presence of IRESes within the VAR 5′UTRs examined. Furthermore, our observations are in agreement with previous studies [19], [20], and imply that additional viral proteins are not required to obtain a basal level of HIV-1 IRES activity in *Xenopus laevis* oocytes.

### Expression of FLuc in the Bicistronic mRNAs is not Due to Cryptic Promoter Activity

**Figure 4:**
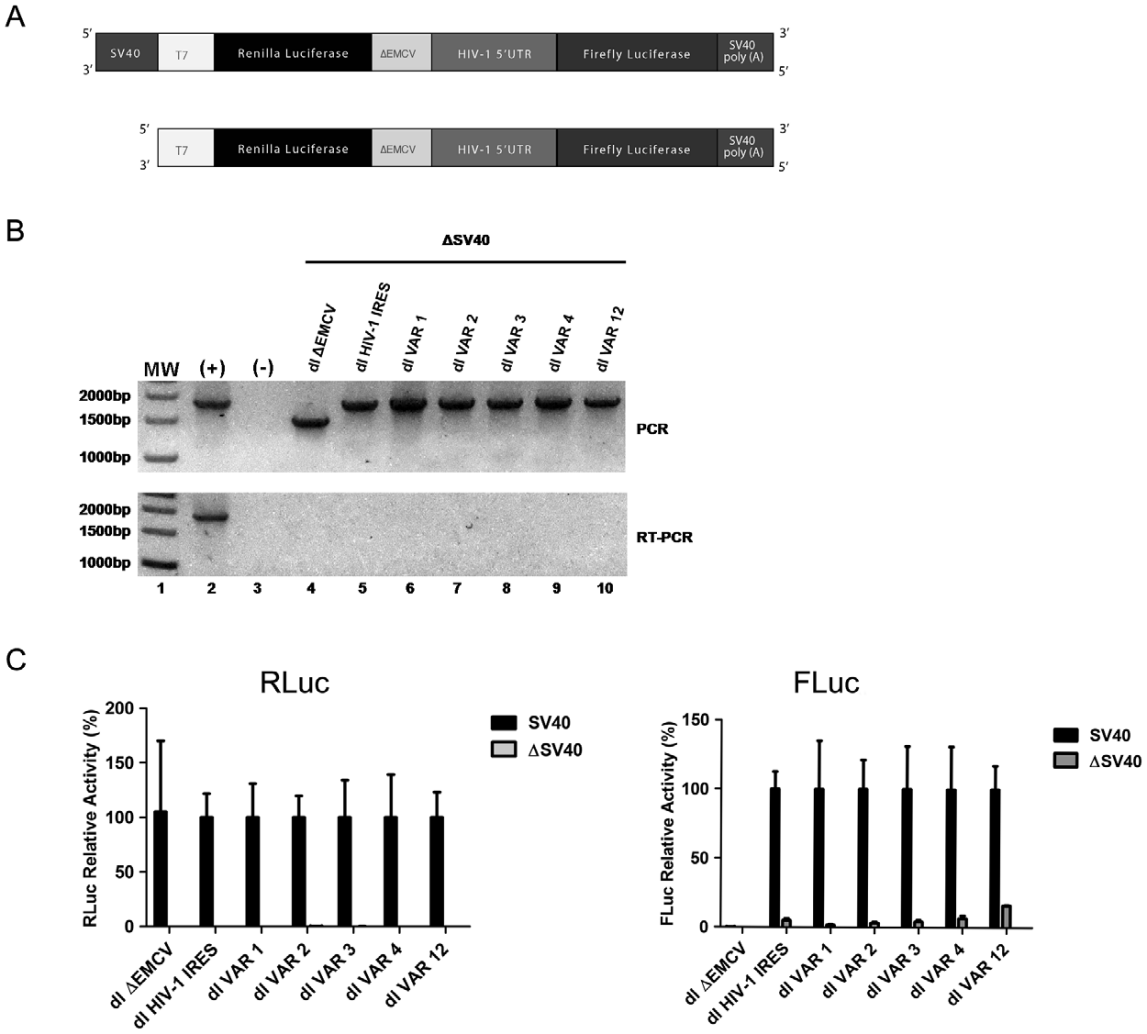
Analysis of a promoterless bicistronic construct containing the HIV-1 5′UTR sequences recovered from clinical samples. (**A**) Schematic representation of the bicistronic constructs. The SV40 promoter from dl ΔEMCV (lane 4), dl HIV-1 IRES, or dl VAR was removed to generate the equivalent promoterless (ΔSV40) vectors. (**B**) HeLa cells were transfected with DNA (200 ng) corresponding to the vectors depicted in (A) as previously described [23]. Total DNA was extracted from transfected cells and the presence of the transfected plasmids was confirmed by PCR (upper panel). The dl HIV-1 IRES plasmid (100 ng) was used as an amplification control. Total RNA was extracted from transfected cells and the presence of transcripts for the ΔSV40-dl ΔEMCV, ΔSV40-dl HIV-1 IRES, or the ΔSV40-dl VAR plasmids was evaluated by a one step RT-PCR designed to detect the bicistronic RNA (depicted in Fig. 2B) [23]. *In vitro* transcribed RNA (100 ng) generated from plasmids dl HIV-1 IRES (lane 2) were used as amplification controls. (**C**) HeLa cells were transfected with either the 200 ng of SV40 or ΔSV40 version of dl ΔEMCV, dl HIV-1 IRES, or the different dl VAR plasmids as previously described [23]. Cells were processed and RLuc and FLuc activities were measured. For each data point the [RLuc/(total protein)] (left panel) and the [FLuc/(total protein)] (right panel) for the SV40 positive plasmids was arbitrary set to 100%. Values are the means +/- SD from three independent experiments.

The firefly luciferase reporter gene has recently been shown to exhibit a cryptic promoter activity that is detectable both in yeast and in mammalian cells [45]. Even though the identified promoter lies within the FLuc coding sequence and is expected to generate shorter mRNAs that do not code for the functional luciferase enzyme [45], we were interested in establishing if possible aberrant transcripts may be influencing the results of the experiments described above (Fig. 1, Fig. 2). For this, we removed the SV40 promoter from the bicistronic constructs (Fig. 4A). In this setting, expression of the downstream transgene would imply that the DNA coding the bicistronic mRNA possesses a cryptic promoter activity that gives rise to at least an RNA species that engenders a functional luciferase enzyme [38]. If so, this aberrant gene expression would be clouding our interpretation of the data [38]. Promoterless bicistronic vectors were transfected into HeLa cells and cells processed 24 h later. In this experiment DNA, RNA, and protein were recovered following previously described protocols [23]. PCR-analysis, confirmed that all cells were positively transfected with the dl vectors (Fig. 4B, upper panel). RNA analysis by RT-PCR [23], confirmed the expression of the bicistronic (RLuc-FLuc) RNA only in cells transfected with the constructs that contained an active SV40 promoter (compare data in Fig. 2 with Fig. 4, lower panel). Furthermore, the analysis of RLuc and FLuc activities confirmed that the reporter proteins were not expressed for any of the dl vectors lacking the SV40 promoter even though DNA was present in cells (Fig. 4C). Detection of the full length bicistronic mRNA is not sufficient to fully discard the production of shorter aberrant RNA species. Nonetheless, the lack of luciferase activity when using the promoterless bicistronic RNAs strongly suggests that if shorter aberrant RNA species are formed their contribution to the overall RLuc and FLuc activity is marginal (Fig. 4C). Results confirm that the full length bicistronic mRNA accounts for most, if not for all, the expression of the luciferase reporters the bicistronic constructs (Fig. 4C).

Careful analysis of the data presented in figure 4C shows low levels of FLuc activity (16%) in the dl VAR 12 lacking the SV40 promoter. Thus, in this particular construct dl VAR 12, we cannot definitively exclude the presence of a cryptic promoter (Fig. 4C). Further experiments are required to absolutely discard this possibility. Nonetheless, and based on the same analysis we exclude the possibility that FLuc activity from the dl VAR 1, dl VAR 2, dl VAR 3, and dl VAR 4 constructs observed in HeLa cells is due to the protein production from an additional transcript encoding FLuc that was generated *via* a cryptic promoter.

### Chemical and Enzymatic Probing of the Variant HIV-1 5′UTRs

**Figure 5:**
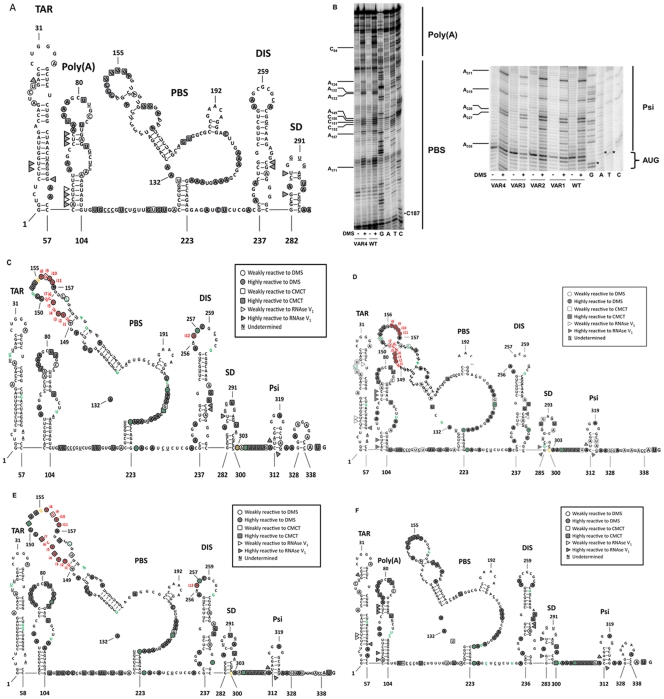
Secondary structure model of the 5′UTR of selected HIV-1 VAR sequences. The HIV-1 5′leader recovered from the dl VAR constructs (followed by 58 nucleotides of fLuc gene) was probed using Dimethyl Sulfate (DMS), N-Cyclohexyl-N0- [N-Methylmorpholino)-ethyl]-Carbodiimide-4-Toluolsulfonate (CMCT) and RNAse V1 as previously described [20]. (A) Secondary structure model of the control (pNL4.3) HIV-1 5′UTR according to DMS, CMCT and RNAse V1 structure probing [6], a key indicating the respective reactivity to the different probes is provided (box). Data included in the figure have been extracted from reference [20]. (B) Typical examples of DMS probing. The HIV-1 5′leader was probed using (+) DMS. Reverse transcription (RT) products were separated on a 8% gel as previously described [12], [20], [48]. Sequencing lanes were also included. Note that DMS induces a premature RT stop one nucleotide before the hit. Therefore the DMS induced stops migrate faster than the corresponding sequence product [12], [20], [48]. The RT pattern of the modified RNA was compared to the profile obtained with an unmodified RNA. Some hits are indicated in the figure. The asterisks on the gel denote the FLuc initiation codon (AUG). Results for VAR 1 (C), VAR 2 (D), VAR 3 (E), and VAR 4 (F) were fitted in a model of the HIV-1 5′ leader [6] as previously described [20]. Numbering in A-F is with respect to clone pNL4.3, here considered the wt sequence. Insertions are indicated in red as independent numbers (iN, were N is the number). Nucleotide changes with respect to clone pNL4.3 are indicated in green. The nucleotide located before a deletion is highlighted in yellow, in this case numbering with respect to pNL4.3 is not altered.

IRES activity is highly dependent on RNA structure [4], [46], [47]. Spurred on by the unexpected translational activity exhibited by variants VAR 1, VAR 2, VAR 3 and VAR 4 relative to the reference pNL4.3 IRES in cells (293%, 409%, 231% and 174%, respectively), we sought to explore the impact of nucleotide variations described in Table 1 on the predicted secondary structure of the respective 5′ UTRs. We recently reported the structure of the HIV-1 5′UTR recovered from the dl HIV IRES construct (nucleotides 1-336 from clone pNL4.3 followed by 58 nucleotides comprising the fluc gene) [20]. The structure was determined by probing RNA using DiMethyl sulfate (DMS), N-Cyclohexyl-N- [N-Methylmorpholino-ethyl]-Carbodiimide-4-Toluolsulfonate (CMCT) and RNAse V1 (Fig. 5A). Using the same experimental approach we probed the 5′UTR recovered from variants dl VAR 1, dl VAR 2, dl VAR 3 and dl VAR 4. CMCT and DMS were used to detect accessible RNA functional groups consisting of single stranded regions, while RNAse V1 revealed stacked or paired nucleotides. Modifications were mapped by reverse transcription using a ^32^P-labeled primer. For each run the reverse transcription (RT) pattern of the modified RNA was compared to the profile obtained with a non-modified RNA. Modifications were classified into two categories according to the relative intensity of the induced stop by comparison with the control. Modifications were classified as “weak” when inducing a 2- to 3-fold increase in intensity of the RT stop, or as “highly reactive” for higher intensities. Typical results using DMS covering the full HIV-1 5′leader are shown (Fig. 5B). Only the strong hits (representing highly reactive modifications) were considered for initial secondary structure modeling using the Mfold algorithm as previously described [12], [48]. The models obtained for all variants (Fig. 5C to 5F), were compatible with the commonly accepted secondary structure model of the HIV-1 5′UTR [6], [20], [49], [50], [51]. Most of the sequence differences can be easily accommodated in the secondary structure since they often lie within loops or unstructured regions (Fig 5C to 5F). Aside from a few point mutations, the main differences between these variants are insertions in the PBS apical loop which appear mostly unstructured as suggested by our chemical and enzymatic probing data (In Fig. 5 compare C, D, E, and F to A). A careful analysis of the probing data reveals three regions that consistently differ in reactivity between the natural HIV-1 variant and the pNL4.3 5′UTR. First, the single strand region lying in between the poly(A) and the PBS structure consistently shows a different pattern in the variants compared to the pNL4.3 5′UTR, this is well exemplified by U_117_ and U_118_. Second, nucleotides A_225_ and G_226_, located 3′ from the PBS in the pNL4.3 sequence, which in the variants HIV-1 5′UTR are consistently two A that are strongly reactive to DMS. Finally, the triplet A_271_G_272_A_273_ bulging out the DIS hairpin are strongly hit in the variants HIV-1 5′UTR although they were not in the pNL4.3 5′UTR. It should be noted however that in a previous “SHAPE” study we found this triplet to be very reactive to the single strand probe 1M7 [20] .

### The Heterogeneous Nuclear Ribonucleoprotein A1 Stimulates HIV-1 IRES Activity

**Figure 6:**
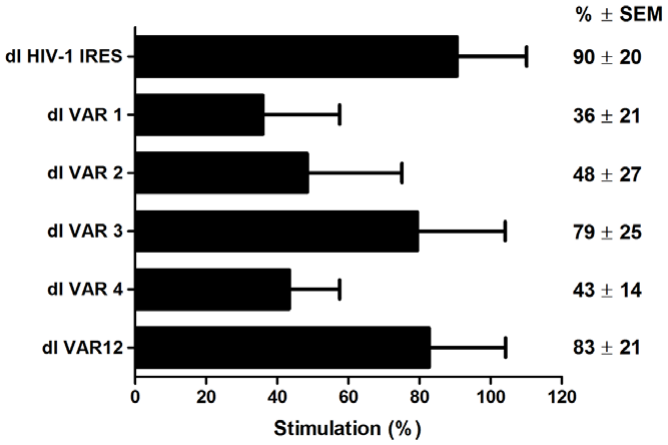
The hnRNPA1 protein enhances translation from the HIV-1 5′UTR. HeLa cells were transfected with either the plasmid expressing hnRNPA1 or the equivalent empty vector in combination with dl HIV-1 IRES or the different dl VAR vectors. Cells were processed and RLuc and FLuc activities were measured. The FLuc/RLuc ratio for each individual bicistronic vector in presence of the empty vector was arbitrary set as the base line. The increase in FLuc/RLuc ratio, which reflects the increase of HIV-1 IRES activity, in presence of the plasmid expressing hnRNPA1 for each individual bicistronic vector is graphed as percentage (%) of stimulation. Values are the means +/- SEM from three independent experiments.

Several events in HIV-1 replication are orchestrated by a diversity of viral and host proteins that interact with each other and with the viral RNA to form HIV-1 ribonucleoprotein (RNP) complexes [52], [53]. Interestingly, these RNP complexes originate in the nucleus and persist in the cytoplasm [52], [53]. Recent studies suggest that the heterogeneous nuclear ribonucleoprotein A1 (hnRNPA1), a protein known to associate with the viral RNA in the nucleus as part of the HIV-1 RNP, plays a role as a positive modulator of HIV-1 IRES activity [18]. Monette et al. (2009) show that hnRNPA1 knockdown reduced HIV-1 IRES activity while protein overexpression in HeLa cells stimulates translation initiation from the HIV-1 5′UTR. These experiments were conducted using the 5′UTR of pNL4.3 this prompted us to explored the effect of hnRNPA1 overexpression on VAR-IRES activity. To evaluate this possibility a plasmid expressing hnRNPA1 [18], was cotransfected with each dl VAR plasmids. In this assay the dl HIV-1 IRES vector was used as a positive control as its response to hnRNPA1 has been documented [18]. After 24 h total proteins were recovered and the FLuc and RLuc activities were measured. The FLuc/RLuc ratio was used as the readout of IRES activity [10], [18], [19], [23], [24]. FLuc/RLuc ratio for each independent dl VAR vector, when co-transfected with the empty control vector was arbitrarily set as the base line IRES activity (Fig. 6). Results for each vector were expressed as percentage of stimulation (%) in respect to its base line (Fig. 6). In agreement with the report of Monette et al. (2003), hnRNPA1 stimulated translational activity from the HIV-1 IRES (90% of additional activity), additionally hnRNPA1 overexpression stimulated translation of all the VAR 5′UTRs (Fig. 6). Therefore, alike the 5′UTR of pNL4.3, all VAR-IRES are responsive to hnRNPA1 overexpression. However, translation stimulation was not equivalent for all VAR 5′UTRs (ranging from 36% to 83%). Strikingly, VAR 1 (36%), VAR 2 (48%) and VAR 4 (43%) 5′UTRs, the IRES elements with the highest activity (Fig. 1B), showed less responsiveness to the overexpression of hnRNPA1 (Fig. 6). Further experiments are required to elucidate the significance of these observations. Nonetheless, results presented in figure 6 confirm that hnRNPA1 is also an IRES transacting factor (ITAF) for the HIV-1 VAR IRESes.

## Discussion

The HIV-1 IRES was identified by cloning the 5**′**UTR of the laboratory adapted HIV-1 infectious recombinant proviral clone NL4.3 (pNL4.3) into the intercistronic region of a dual luciferase (dl) reporter construct [10]. The dl reporter construct contained the *Renilla* luciferase gene (RLuc) upstream and the firefly luciferase gene (FLuc) downstream. To ensure that the two cistrons were independently translated, a defective encephalomyocarditis virus IRES (?EMCV), known to inhibit ribosome reinitiation and read-through [10], [25], was inserted upstream of the HIV-1 5**′**UTR [10]. In this experimental setting the translational activity of the HIV-1 5**′**UTR was monitored using FLuc activity as the readout, while the RLuc reporter gene served as an upstream translational control. This approach demonstrated that, when in the context of a bicistronic mRNA the HIV-1 5**′**UTR of pNL4.3 was capable of driving efficient translation initiation [10]. Most recently [14], the presence of a competent IRES was also reported within the 5**′**UTR of the CXCR4 *(*X4*)-*tropic primary isolate HIV-LAI [14]. Since the discovery of the HIV-1 IRES element, a number of additional studies have focused on understanding the mechanisms underlying its function [9], [17], [18], [19], [20]. However, to date all studies regarding the HIV-1 IRES have been limited to the 5**′**UTR of pNL4.3 or pLAI, raising the question as to the significance and validity of these findings in the context of naturally circulating viral variants. Here we provide evidence that HIV-1 5**′**UTR sequences recovered from clinically sourced RNA isolates do indeed harbor an IRES, suggesting that the ability of HIV-1 mRNA to drive cap-independent translation initiation is likely to be widespread if not ubiquitous and is not restricted to the laboratory adapted HIV-1 clone pNL4.3 or to the single primary HIV-1 isolate, pLAI.

The full length mRNAs of the HIV-1 clone pNL4.3 and pLAI can initiate translation through a canonical cap-dependent mechanism [7], [8], [9], or by an alternative IRES-dependent mechanism [9], [10], [11], [12], [13], [14]. In this new study the translational activity of the 5**′**UTR of viral RNA isolated from HIV-1 infected individuals (VAR 5**′**UTRs) was evaluated in the context of a bicistronic mRNA [10], [14], [22]. Cap-independent translation initiation from the VAR 5**′**UTRs was demonstrated in HeLa cells and in *Xenopus laevis* oocytes (Fig. 2, Fig. 3 and Fig. 4). The notion that expression of the second cistron in the bicistronic constructs may be the result of alternative splicing or due to cryptic promoter activity was also ruled out (Fig. 2, Fig. 3, and 4). Together, data suggest that the 5**′**UTR of the HIV-1 mRNAs isolated from clinical samples enables IRES-mediated translation initiation. Additionally, data presented in figures 1 and 2, confirm previous observations that active translation from the HIV-1 IRES in both HeLa cells and in *Xenopus laevis* oocytes does not rely on additional viral proteins [19], [20].

The 5**′**UTR recovered from natural viral variants exhibited extensive sequence diversity when compared to the control sequence (Table 1). Furthermore, translational activity from some of the HIV-1 VAR 5**′**UTRs was enhanced substantially when compared to the control pNL4.3 5**′**UTR (Fig. 1 and Fig 3) and yet these presented a number of nucleotide variations within the PBS-stem loop (Table 1), a structural element known to be required for IRES-mediated translation initiation [10]. These observations prompted us to evaluate possible structural differences between the pNL4.3 5**′**UTR [10], and the VAR 5**′**UTRs recovered from clinical samples (Fig. 5). In summary, data showed that the only differences between the VAR 5**′**UTRs and the pNL4.3 5**′**UTR lied within single stranded RNA regions (Fig 5C to 5F), being the only exception the U5 region lying in between the poly(A) and the PBS structure, which has been predicted to be paired with the nucleotides surrounding the initiation codon [49], [51], [54]. The enhanced translational activities exhibited by some of the VAR 5**′**UTR could therefore be attributed to a better accessibility to the initiation codon region. However, we could not see an increase in reactivity in this particular region (Fig. 5). Overall, enhanced translational activities exhibited by some of the VAR 5**′**UTRs cannot easily be ascribed to specific RNA structural differences when compared to the control pNL4.3 5**′**UTR. Instead it may be that nucleotide differences impact on the ability of the RNA to recruit protein factors required for optimal IRES activity. [20]. Considerable work has been directed toward the identification of the protein factors participating in IRES mediated translation initiation [4], [55]. Translation driven by some viral IRESes precludes the need for certain host eIFs (differing according to the group of IRESes under study), yet they often require additional host proteins, the IRES *trans*-acting factors (ITAFs) [4], [46], [55], [56]. Thus, enhanced IRES activity exhibited by variant 5**′**UTRs, which, as shown herein share RNA secondary structure similarity with the pNL4.3 5**′**UTR (Fig. 5), may be due to an altered capacity of these variant RNAs to bind specific IRES activator or inhibitor proteins [17], [18], [19], [20]. We sought to evaluate this possibility by analyzing the effect of hnRNPA1 on translation driven by the variant 5**′**UTR. A previous study defined hnRNPA1 as an ITAF for the pNL4.3 IRES [18]. As shown in figure 6, translation driven by all variant 5**′**UTRs was enhanced in presence of hnRNPA1, observation that is consistent with the findings of Monette et al. (2009). However, responsiveness to hnRNPA1 over expression was not equivalent for all VAR 5**′**UTRs (Fig. 6). Further hnRNPA1-RNA interaction experiments are required to rule out if the lack of responsiveness to hnRNPA1 overexpression is due to an altered capacity of these variant RNAs to bind the protein. In any case results suggest that hnRNPA1 is also an ITAF for the VAR-IRESes. It should be noted that several other proteins, including the human embryonic-lethal abnormal vision (ELAV)-like protein HuR, the eukaryotic translation initiation factor 5A (eIF5A), the human Rev-interacting protein (hRIP), and the DEAD (Asp-Glu-Ala-Asp) box polypeptide 3 (DDX3), have been identified as modulators of HIV-1 IRES activity [17], [19], [20]. Furthermore, and based on the report of Vallejos et al. (2011), the list of cellular proteins that modulate HIV-1 IRES activity is expected to increase [20].

As for most retroviral IRES elements, the biological significance of the HIV-1 IRES within the viral context remains obscure [16]. Furthermore, several studies using monocistronic RNAs suggest that translation initiation from the HIV-1 mRNA is mostly cap-dependent [7], [8], [9]. These studies, conducted under conditions known to favor cap-dependent translation initiation, do not exclude the existence of an IRES within the 5**′**UTR [8], [9], [20], [57]. These studies do however shed light on the mechanism of translation initiation used by an important subpopulation of viral mRNAs. HIV-1 mRNAs are composed of a mixture of monomethylated capped (7-methylguanosine, 7mG-) and trimethylated (TMG)-capped mRNAs [58]. TMG-capped-RNAs are known to translate poorly [59], due to the inefficient recognition of TMG-caps by the eukaryotic initiation factor 4E (eIF4E), the cap-binding protein [60], [61], [62], [63]. The recognition of the 5′ cap by eIF4E is the rate-limiting step in the cap-dependent ribosome recruitment to mRNAs [1], [3], [55]. Therefore, TMG-HIV-1 RNAs are likely to exhibit inefficient cap-dependent translation initiation, thus reducing expression of the viral structural proteins. Yet, in sharp contrast to the above prediction, TMG-capping of viral mRNAs increases the expression of HIV-1 structural proteins [58]. The mechanism by which these TMG-capped viral mRNAs recruit the translational machinery remains unevaluated, yet it is tempting to speculate that translation initiation from the TMG-HIV-1 RNAs is mostly IRES-dependent. This possibility would partially justify why the HIV-1 full length mRNA requires an IRES. An additional set of reports also advocate in favor of the requirement of a cap-independent initiation mechanism for the HIV-1 mRNA [64], [65], [66], [67], [68], [69]. IRES-mediated translation initiation has been proposed to allow the viral mRNA to bypass the constraints of global cellular translation repression that normally targets cap-dependent translation initiation [1], [3], [55]. In the case of the HIV-1 mRNA, IRES-mediated initiation would support viral protein synthesis during the G2/M phase of the cell cycle and during osmotic stress [10], [14], [18]. Additionally, cap-independent translation initiation would ensure synthesis of HIV-1 structural proteins during the late stages of the replication cycle, when the eukaryotic initiation factor eIF4G and the poly(A) binding protein (PABP), both required for cap-dependent translation initiation, are targeted by the HIV-1 protease [64], [65], [66], [68], [69]. In consequence, harboring an IRES would allow the HIV-1 mRNA to overcome translational constrains that specifically target cap-dependent translation initiation imposed in part by the viral replication cycle itself.

## Materials and Methods

### Viral RNA Purification

Surplus total RNA, extracted using the High Pure Viral Nucleic Acid kit (Roche Diagnostics, applied science, Mannheim, Germany) from the serum of HIV-1 infected patients, normally discarded upon viral load determination, was randomly pooled and used in this study as the source of viral RNA. Pooled RNA samples were provided by the Laboratorio de Infectología, Facultad de Medicina, Pontificia Universidad Católica de Chile.

### Plasmid Construction

The dl ΔEMCV and the dl HIV-1 IRES plasmids were previously described [10], [25]. For the generation of the bicistronic vectors dl VAR, the 5**′**UTR of natural variants were recovered from the randomly pooled viral RNA extracts by RT-PCR using the SuperScript™III one step RT-PCR system with platinum® Taq DNA polymerase (Invitrogen, Life Technologies Corporation, Carlsbad, California, USA) using the primers HIV1-F (5**′**-CACGAATTCGGTCTCTCTG-3′) and HIV1-R (5′-CCATGGTCTCTCTCCTTC-3′). All new sequences identified in this study can be found in GenBank (JN642552 to JN642564). The dl VAR vectors were generated as previously described [10], in brief the amplicon was digested with EcoRI and NcoI (both restriction sites added by PCR) and ligated, using a triple ligation strategy, with the 5248 bp-EcoRI/XbaI and 1656 bp -NcoI/XbaI fragments of the previously digested dl HIV-1 IRES [10]. To generate plasmids without the SV40 mammalian promoter, the bicistronic vectors were digested with StuI and MluI (Fermentas), treated with the *E. coli* DNA Polymerase I Klenow fragment (Fermentas) to generate blunt ends, and ligated using T4 DNA ligase (Fermentas). All plasmids were confirmed by sequencing (Macrogen Corp, Rockville, MD, USA).

### Cell Culture

HeLa cells, similar to those used in previous studies [10], [19], [20], kindly provided by Dr. Nahum Sonenberg (McGill University, Montreal, Canada), were cultured in Dulbecco’s modified Eagle’s medium (Gibco-BRL) with 50 U/mL of penicillin-streptomycin (HyClone) and 10% fetal bovine serum (HyClone) at 37°C in a 5% CO_2_ atmosphere.

### DNA Transfection

Cells were seeded at 10^5^cell/well in 12-well plates. DNA transfection was performed at 60% confluency by the JetPei system (Polyplus-transfection SA, Illkirch, France) according to the manufacturer’s protocol. For the hnRNPA1 over expression experiments, the well characterized hnRNPA1 expression plasmid (125 ng) [18], kindly provided by Dr. Andrew Mouland (Lady Davis Institute for Medical Research-Sir Mortimer B. Davis Jewish General Hospital, Montreal, Canada) and Benoit Chabot (University of Sherbrooke, Sherbrooke, Canada) or the empty vector DNA (125 ng) were transfected in cells together with the bicistronic vectors (200 ng). After 24 hours, the culture medium was removed and the cells were lysed with the passive lysis buffer as described in the DLR™ Assay System manual (Promega, Madison, WI, USA). Luciferase activities were measured using the DLR^TM^ Assay System (Promega) according to the manufacturer’s instructions on a Sirius Single Tube Luminometer (Berthold Detection Systems GmbH, Pforzheim, Germany).

### RNA, DNA Extraction, PCR, RT-PCR and RT-qPCR

Cells were washed with phosphate-buffered saline (PBS, 137 mM NaCl, 2.7 mM KCl, 4.3 mM Na_2_HPO_4_·7H_2_O, 1.4 mM KH_2_PO_4_, pH 7.4) at 4°C and lysed in 100 µL of RLNa buffer (10 mM Tris–HCl (pH 8.0), 10 mM NaCl, 3 mM MgCl_2_, 1 mM DTT, 0.5% NP40 and 10 U/mL of RiboLock™ RNase Inhibitor (Fermentas) for 2 min on ice. Supernatants were mixed with 1 mL of TRIzol® Reagent (Invitrogen) and total RNA was extracted according to the manufacturer’s instructions. Total RNA was resuspended in 20 µL of nuclease-free water and treated using the DNA-free™ kit (Applied Biosystems/Ambion, Austin, TX, USA) according to the manufacturer’s protocol. The integrity of the RNA obtained was confirmed by electrophoresis on 1% agarose gels and quantified by spectrophotometry (NanoDrop Technology). DNA was extracted from cells using the EZNA^TM^ kit (Omega Bio-Tek, Inc., GA, USA) according to the manufacturés protocol. DNA concentration was determined by spectrophotometry (NanoDrop Technology).

The PCR assay was conducted using primers p2anti (5**′**-TCTCTTCATAGCCTTATGCAGTTG-3**′** ) and Pforluc (5**′**-CATGACTTCGAAAGTTTATGATC-3**′** ), 100 ng of total DNA and the Go Taq®Green Master mix (Promega), according to the manufacturer’s protocol. The RT-PCR assay was carried out with SuperScript™ III One-Step RT-PCR System using Platinum® Taq DNA polymerase (Invitrogen) according to the manufacturer’s protocol, using 1 µg of RNA and the primers described above.

The RT-qPCR assay was conducted using the primers RLucS (5**′**-AGGTGAAGTTCGTCGTCCAACATTATC-3**′** ) and RLucAS (5**′**-GAAACTTCTTGGCACCTTCAACAATAGC-3**′** ) for RLuc amplification (193bp); or FLucS (5**′**- ACTTCGAAATGTCCGTTCGG-3**′** ) and FLucAS (5**′**- GCAACTCCGATAAATAACGCG-3**′** ) for FLuc amplification (135bp), using the Brilliant® II SYBR® Green QRT-PCR Master Mix KIT (Stratagene, Agilent Technologies Company, Santa Clara, CA, USA). The amplification reactions were conducted under the following conditions: 30 min at 45°C for RT step followed by 10 min at 94°C, continued by 40 cycles of 20s at 94°C, 20s at 60°C and 30s 72°C. The melting curve was performed between 60°C and 90°C to verify the reaction specificity. For each experiment, the amount of RNA-RLuc and RNA-FLuc for each bicistronic RNA was determined (pmoles) based on an individual standard curve constructed using *in vitro* transcribed RNA as a template. Values were corrected considering the amplification efficiency of each couple of primers. The RNA-FLuc/RNA-RLuc ratio was used to compare the amount of bicistronic RNA in each transfection assay.

### *In vitro* Transcription

Capped RNAs were synthesized using the mMESSAGE mMACHINE High Yield Capped RNA Transcription Kit (Applied Biosystems/Ambion, Austin, TX, USA) and polyadenylated using the Poly(A) Tailing kit (Applied Biosystems/Ambion) according to the manufacturer’s protocol. Uncapped RNA was synthesized by *in vitro* transcription conducted in a final volume of 100 µL using T7 RNA polymerase, 5 mM DTT, 5 mM rNTP’s, 1X transcription buffer (40 mM Tris-HCl pH8.0, 25 mM MgCl_2_, 1 mM spermidine), 0.04 U RNase inhibitor (Applied Biosystems/Ambion), and incubated for 120 min at 37°C. To eliminated the DNA template, 10 µL of DNAse RQ1 (Promega) was added, and further incubated for 20 min at 37°C. The RNA was precipitated using 2.5 M LiCl (−20°C for 30 min), centrifuged at 16000×g (30 min at 4°C), washed with 70% ethanol and resuspended in 50 µL of nuclease-free water. RNA concentrations were determined spectrophotometrically (NanoDrop Technology) and RNA integrity was monitored by electrophoresis on denaturing agarose gels.

### Oocyte Harvesting and RNA Microinjection

Oocytes were isolated from *Xenopus laevis* ovarian fragments by manual dissection as previously described [70]. Oocytes were incubated at 15°C for 24 h in standard Barth’s solution supplemented with 10 IU/l penicillin–streptomycin, and 2 mM pyruvate [70]. To evaluate viral IRES activity in oocytes, 6.25 ng of *in vitro* transcribed capped and polyadenylated RNA was microinjected into each oocyte with glass micropipettes calibrated to deliver a final volume of 50 nl [71], [72]. After 24 h oocytes were lysated in Passive Lysis buffer (Promega), centrifuged at 16,000x g for 5 min, and 1-5 µl of the supernatant was used in the detection assay using the DLR™ Assay System kit (Promega).

### RNA Probing

The secondary structure of the HIV-1 5**′**UTR *in vitro* transcribed RNAs (10 pMoles), which included the 5**′** leader of HIV-1 (pNL4.3) and the first 58 nt of fluc open reading frame (recovered from the dl HIV-1 IRES and dl VAR plasmids using a primer T7HIVF 5**′**-CCATATGTAATACGACTCACTATAGGTCTCTCTGGTTAGA-3**′** and Fluc30bp 5**′**-CATCTTCCAGCGGATAGAATG-3**′** ), was probed using DiMethyl Sulfate (DMS, Across Organics), N-cyclohexyl-N-[N-methylmorpholino)-ethyl]-carbodiimid-4-toluolsulfonate (CMCT, Merck) and RNAse V1 (Applied Biosystems/Ambion) as described previously [12], [20], [48]. The RNAs were resuspended in 30 µl of 80 mM HEPES pH 7.5 (or 50 mM borate potassium pH 8.0 for CMCT), denatured for 2 min at 80°C, and then 2 µL of 3M KCl and 2 µL of 40 mM MgCl_2_ were added. Following a10 min incubation at 30°C, DMS (0.2 mM final), CMCT (25 mM final) or RNAse V1 (0.01 or 0.025 U) was added and the mixture was incubated for 5 min (10 min for CMCT). Mock controls, where the chemical was replaced by water, were also included. The modification reaction was stopped on ice by addition of 10 µg of total yeast tRNA, and precipitated on dry ice with ethanol and 5 M ammonium. RNA was then resuspended in 0.5 M ammonium acetate, ethanol precipitated and resuspended in 6 µL of nuclease-free water. Modifications were revealed by reverse transcription (AMV RT, Promega) using ^32^P-labelled primer (Fluc30bp, HIV1-336R: 5**′**-TTTGAAAAACACGAATTCGGTCTCTCTG-3′, 100pbHIV-1: 5**′**-ACTTTGAGCACTCAAGGCAAG-3**′** , 200pbHIV-1: 5**′**-TTCGCTTTCAAGTCCCTGTTC-3**′** ) according to the manufacturer’s instructions (Promega). Reverse transcription products were resolved on an 8% denaturing urea-polyacrylamide gel, the resulting gel was scanned on a Typhoon Trio Variable Mode imager (Amersham Biosciences). The relative proportion of each product was determined by drawing profiles using Multi Gauge V3 software (Fujifilm).
